# Cardiopulmonary fitness and quality of life improvements in atrial fibrillation patients following exercise-based cardiac rehabilitation: meta-analysis of randomized control trials

**DOI:** 10.1016/j.clinsp.2026.100907

**Published:** 2026-03-20

**Authors:** Zhen Zhang, Jialing He, Yinlan Hu, Shuyao Wang, GuoShu Yang, Duan Luo, Xu Bao, Guijun He, Xianchen Yang, Hanxiong Liu

**Affiliations:** a Department of Cardiology, The Affiliated Hospital of Southwest Jiaotong University, The Third People's Hospital of Chengdu, Cardiovascular Disease Research Institute of Chengdu, China; b Physical Examination Department, Modern Hospital of Sichuan, China

**Keywords:** Atrial fibrillation, Exercise rehabilitation, Cardiopulmonary fitness, Quality of Life

## Abstract

**Background:**

In this meta-analysis, the authors aimed to provide a comprehensive overview of the benefits of exercise and its potential implications in the management of atrial fibrillation.

**Method:**

The authors conducted searches in PubMed, MEDLINE, EMBASE, and Cochrane databases using the terms “atrial fibrillation”, “exercise”, “cardiac rehabilitation”, or “physical activity”. The endpoints included the 6-Minute Walk Test (6MWT), maximal Oxygen uptake (VO_2_max) measured by Cardiopulmonary Exercise Testing (CPET), maximal power measured by CPET, resting Heart Rate (HR), Left Ventricular Ejection Fraction (LVEF), data from the 36-Item Short Form Survey Instrument (SF-36), and AF recurrence post-ablation.

**Results:**

The present analysis included 11 randomized control trials published in the past 15-years, involving a total of 1251 patients. The pooled analysis showed significant increases in VO_2_max, 6MWT, and maximal power in AF patients post-exercise (-3.05, 95 % CI: [-4.79, -1.3], *I^2^* = 84 %, *p* < 0.0001; -42.07, 95 % CI: [-56.22, -27.93], *I^2^* = 0 %, *p* < 0.00001; -16.39, 95 % CI: [-20.76, -12.01], *I^2^* = 72 %, *p* < 0.00001, respectively). Sensitivity analysis results remained consistent but significantly reduced heterogeneity. The authors found that the recurrence rate was significantly lower in patients who exercised (RR = 0.76, 95 % CI: [0.62, 0.95], *I^2^* = 0 %, *p* = 0.01). Quality of life was assessed using the SF-36 scale, which includes eight domains. The pooled results are as follows: physical function (WMD = -6.46, 95 % CI: [-10.48, -2.44], *I^2^* = 52 %, *p* = 0.002), role physical (WMD = -13.02, 95 % CI: [-16.68, -9.36], *I^2^* = 32 %, *p* < 0.00001), general health (WMD = -7.80, 95 % CI: [-12.35, -3.25], *I^2^* = 62 %, *p* = 0.0008), bodily pain (WMD = -3.54, 95 % CI: [-6.82, -0.26], *I^2^* = 0 %, *p* = 0.03), vitality (WMD = -8.34, 95 % CI: [-14.31, -2.36], *I^2^* = 74 %, *p* = 0.006), social function (WMD = -6.44, 95 % CI: [-12.17, -0.72], *I^2^* = 71 %, *p* = 0.03), role emotional (WMD = -12.18, 95 % CI: [-16.46, -7.90], *I^2^* = 0 %, *p* < 0.00001), and mental health(WMD = -6.79, 95 % CI: [-11.69, -1.88], *I^2^* = 72 %, *p* = 0.007). Sensitivity analysis resulted in consistent outcomes with heterogeneity reduced to 0 % for all domains.

**Conclusion:**

The present study found that exercise rehabilitation benefits patients with atrial fibrillation. Exercise can improve quality of life and possibly reduce recurrence. While it enhances cardiorespiratory fitness (VO_2_max, 6MWT, maximal power), it does not significantly improve ejection fraction or heart rate.

## Highlights

• Exercise improves cardiorespiratory fitness.
• Enhance QoL.
• Reduce AF recurrence.

## Introduction

Atrial Fibrillation (AF) is a prevalent cardiac arrhythmia characterized by irregular, rapid electrical activity of the atria, often accompanied by symptoms such as palpitations and dyspnea. AF increases cardiovascular disease and stroke risk, diminishes quality of life, and heightens mortality rates. With global demographic shifts toward an aging population and rising chronic disease burdens, AF has emerged as a major challenge in cardiovascular disease management.^1^ In addition to pharmacological and surgical interventions, exercise rehabilitation plays an important role as an alternative therapy in controlling AF symptoms and preventing complications.

Exercise is increasingly recognized as integral to cardiac rehabilitation across various cardiovascular conditions, including ischemic heart disease, heart failure, hypertension, and peripheral arterial disease.^2^ The 2020 European Society of Cardiology (ESC) guideline for the diagnosis and management of AF underscores the importance of cardiorespiratory exercise testing and cardiorespiratory fitness, stating that “patients should be encouraged to undertake moderate-intensity exercise and remain physically active to prevent AF incidence or recurrence”.^3^ And guidelines emphasize the primary and secondary preventive effects of physical activity and provide specific guidance for those with risk factors of cardiovascular disease.^2^ In this context, the authors conducted a meta-analysis to synthesize findings on the physiological and quality-of-life changes in AF patients following exercise-based rehabilitation. The authors’ aim is to provide a comprehensive overview of the benefits of exercise and its potential implications in the management of AF.

## Method

### Study selection

The authors searched PubMed, MEDLINE, EMBASE and Cochrane databases (inception to April 4st, 2024) using the terms “atrial fibrillation”, “exercise” OR “cardiac rehabilitation” OR “physical activity”. In addition, the authors reviewed the reference lists of retrieved studies and major conference proceedings. Any article that met the criteria listed in the following section was retrieved. When groups published multiple reports with overlapping cohorts, the most recent study was included.

The inclusion criteria were as follows:

1. The study population was patients with atrial fibrillation.
2. Randomized Controlled Trial (RCT).
3. literature published in recent 15-years.
4. Includes pre- and post-exercise data separately from patients (details in data extraction part).
5. Studies directly report mean value and Standard Deviation (SD), or have sufficient data to calculate them.

The Cochrane Collaboration tool was utilized for assessing the risk of bias.^4^ Data extraction and quality assessment of the included studies were performed independently by three authors (JLH, YLH, and BX). Consensus among all three reviewers was required to finalize the classification of the studies. Specific references for the data used in the analysis are provided within the manuscript and are accessible to all researchers.

### Data extraction

Three authors independently conducted database searches and reached a consensus on the inclusion of selected studies. Data extraction and preparation of this article followed the recommendations of the PRISMA group.

The extracted data included information on study type, year of publication, type of exercise (supervised or home-based), exercise frequency, intensity, duration, follow-up duration, and pre- and post-exercise measurements. Specific measurements and endpoints were the 6-Minute Walk Test (6MWT), maximal Oxygen uptake (VO_2_max) measured by Cardiopulmonary Exercise Test (CPET), maximal power measured by CPET, resting Heart Rate (HR), Left Ventricular Ejection Fraction (LVEF), data from the 36-Item Short Form Survey Instrument (SF-36), and AF recurrence post-ablation.

### Statistical analysis

All analyses were conducted using Review Manager, version 5.4. Weighted Mean Differences (WMD) with corresponding 95 % Confidence Intervals (95 % CI) were used as the effect size for continuous variables. Categorical variables were reported as pooled Risk Ratios (RR). Statistical heterogeneity for each outcome of interest was assessed using the p-value for the *Q* statistic and the *I^2^* statistic. Heterogeneity was considered low if *I²* < 25 %, moderate if 25 % ≤ *I^2^* ≤ 75 %, and high if *I^2^* > 75 %. Summary estimates were presented in forest plots. A fixed-effects model was used if *I^2^^〈^* 50 %, whereas a random-effects model was employed if *I^2^* > 50 %. In cases of substantial between-study heterogeneity, subgroup analyses were conducted to identify potential sources of heterogeneity. Sensitivity analysis was performed by stepwise exclusion of one study at a time to determine the impact of individual studies on the overall outcomes.

## Result

**Fig. 1:**
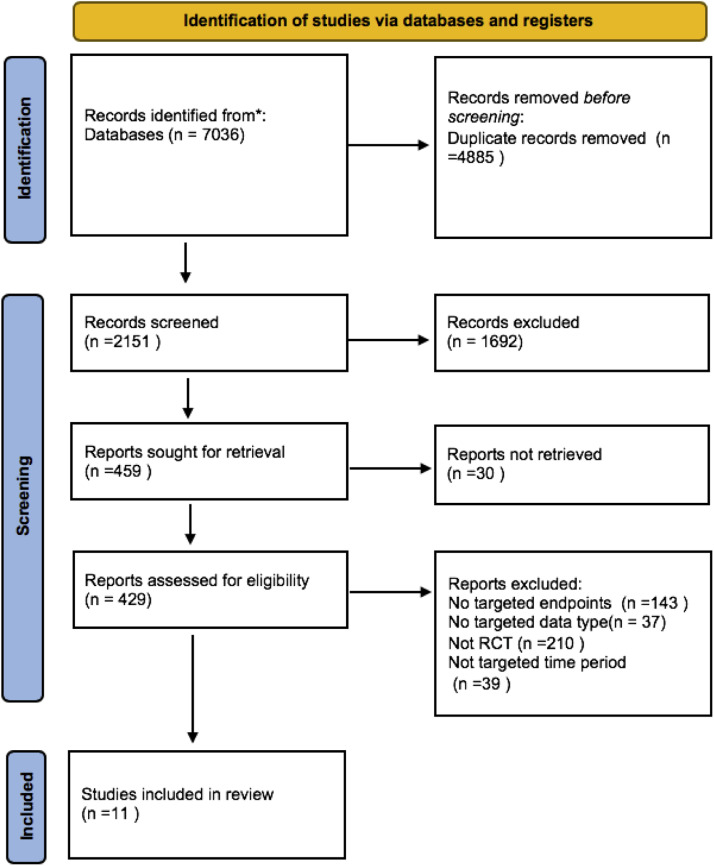
Flowchart for study selection.

The systematic search identified a total of 7036 records. Following the deduplication process, 2151 studies remained for title and abstract screening. Of these, 459 articles were selected for full-text review. Based on the inclusion criteria and a rigorous screening process, 11 studies were ultimately included. All 11 of these studies employed randomization methods. The whole involved population was 1251. The specific screening process is detailed in Fig. 1, while the quality evaluation of the included studies is presented in Supplementary Figure 1. The characteristics of the included studies are displayed in Table 1.

**Table 1:** Characteristics of involved studies.

**Study**	**Nation**	**Intervention**	**Control**	**Population**		**Ablation**	**AF type**	**Follow-up**	**Rehabilitation type**	**Rehabilitation duration**	**Exercise type**	**Exercise frequency**	**Exercise intensity**
				**Intervention**	**Control**								
Chen 2019	China	Exercise rehabilitation	Usual care	62	60	Receive ablation	Paroxysmal	12-months	Supervised rehabilitation	12 months	Aerobic exercise	3‒4 times/week	HR <160 bpm
Risom 2020	Denmark	Physical exercise and psychoeducational consultations plus usual care	Usual care	105	105	Receive ablation	Paroxysmal/ Persistent	12 and 24 months	Supervised rehabilitation, some followed by home-based training	12 weeks	NA	3 times/week	NA
Risom 2016	Denmark	Physical exercise and psychoeducational consultations plus usual care	Usual care	105	105	Receive ablation	Paroxysmal/ Persistent	6 months	Supervised rehabilitation, some followed by home-based training	12 weeks	NA	NA	NA
Osbak 2012	Denmark	Exercise rehabilitation	Usual care	25	24	Receive ablation	Permanent	4 months	Supervised rehabilitation	12 weeks	Aerobic exercise:	3 times/week	Borg scale scores 14‒16
Joensen 2019	Denmark	Exercise rehabilitation and education	Usual care	30	58	Receive ablation	Paroxysmal/ Persistent	3, 6 and 12 months	Supervised rehabilitation	12 weeks	Aerobic exercise	2 times/week	Borg scale scores 14‒16
Kato 2019	Japan	Exercise rehabilitation	Usual care	30	31	Receive ablation	Persistent	6 months	Supervised rehabilitation	6 months	Aerobic and anaerobic exercise	2‒3 times/week	Moderate (no definition)
Elliott 2023	Australia	Exercise rehabilitation	Usual care	60	60	Without ablation	Paroxysmal/ Persistent	12 months	Supervised and home-based	6 months	Aerobic exercise	1‒2 times/week	85 %‒90 % heart rate reserve
Cai 2022	China	Home-based mobile application-guided and tele-monitored cardiac rehabilitation	Usual care	50	50	Receive ablation	Paroxysmal/ Persistent	4 months	Supervised and home-based	12 weeks	Aerobic exercise:	5 times/week	NA
Borland 2020	Sweden	Physiotherapist-led exercise-based cardiac rehabilitation	Physical activity on prescription	46	50	NA	Persistent	3 months	Supervised and home-based	3 months	Aerobic exercise:	Intervention group 2 times/week; control group 4 times/week	Borg scale scores 6‒20
Wahlström 2020	Sweden	Medi Yoga	No Medi Yoga	38	38	NA	Paroxysmal	12 weeks	Supervised rehabilitation	12 weeks	Medi yoga	Once a week	NA
Nguyen 2020	Netherland	Exercise rehabilitation	Usual care	80	39	NA	Persistent	12-month	NA	9‒11 weeks	Aerobic exercise:	2‒3 times/week	Moderate intensity (3–6 METs); vigorous intensity (>6 METs).

### Changes in cardiopulmonary fitness

**Fig. 2:**
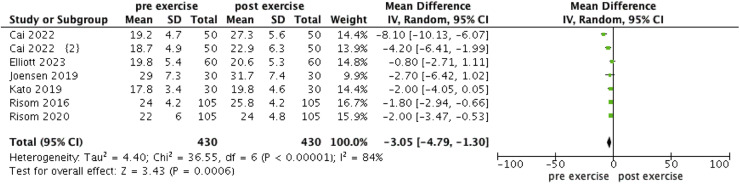
Forest plot of VO_2_max.

**Fig. 3:**
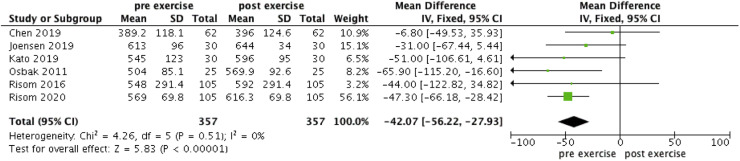
Forest plot of 6MWT.

**Fig. 4:**
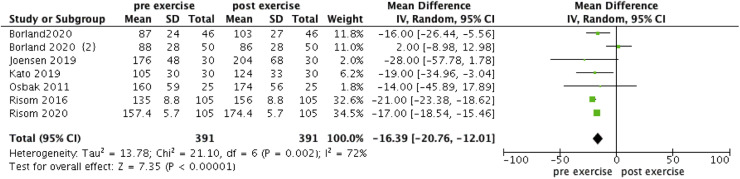
Forest plot of maximal power.

The forest plot illustrating the change in VO_2_max, 6MWT and maximal power in AF patients engaging in exercise is presented in Fig. 2, Fig. 3, Fig. 4. VO_2_max, 6MWT, and maximal power all showed significant increases post-exercise (WMD = −3.05, 95 % CI: [−4.79, −1.3], *I^2^* = 84 %, *p* < 0.0001; WMD = −42.07, 95 % CI: [−56.22, −27.93], *I^2^* = 0 %, *p* < 0.00001; WMD = −16.39, 95 % CI: [−20.76, −12.01], *I^2^* = 72 %, *p* < 0.00001 respectively).

In the meta-analysis of VO_2_max, excluding the study by Cai et al. reduced the heterogeneity to 0 % (WMD = −1.76, 95 % CI: [−2.5, −1.02], *I^2^* = 0 %, *p* < 0.00001). Similarly, in the meta-analysis of maximal power, excluding the study by Borland et al. reduced the heterogeneity to 40 % (WMD = −18.17, 95 % CI: [−19.33, −16.89], *I^2^* = 40 %, *p* < 0.00001).

**Fig. 5:**
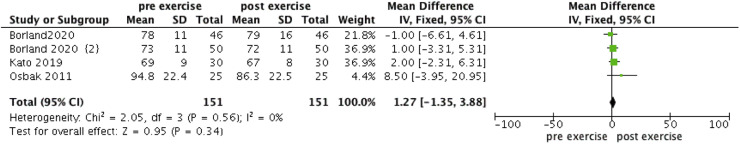
Forest plot of resting heart rate.

**Fig. 6:**
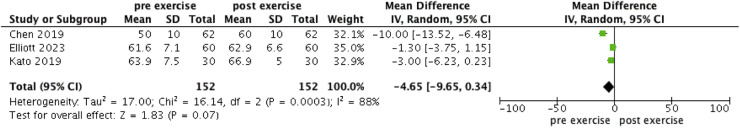
Forest plot of left ventricle ejection fraction.

Additionally, no significant differences were observed in Heart Rate (HR) and Ejection Fraction (EF) following exercise, as shown in Fig. 5, Fig. 6 (HR: WMD = 1.27, 95 % CI: [−1.35, 3.88], *I^2^* = 0 %, *p* = 0.34; EF: WMD = −4.65, 95 % CI: [−9.65, 0.34], *I^2^* = 88 %, *p* = 0.07, respectively).

### AF recurrence after ablation

**Fig. 7:**
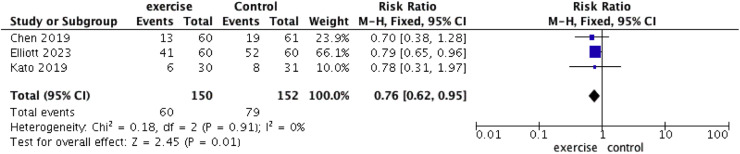
Forest plot of recurrence after ablation.

Three studies reported on the recurrence rates in patients who underwent exercise training after catheter ablation (Fig. 7). The authors found that the recurrence rate was significantly lower in patients who exercised, with very low heterogeneity (RR = 0.76, 95 % CI: [0.62, 0.95], *I^2^* = 0 %, *p* = 0.01).

### Changes in quality of life

A total of six studies reported changes in the quality of life, as estimated by the SF-36, in patients with Atrial Fibrillation (AF) after participation in exercise. The results (listed in Table 2) are as follows: physical function (WMD = −6.46, 95 % CI: [−10.48, −2.44], *I^2^* = 52 %, *p* = 0.002), role physics (WMD = −13.02, 95 % CI: [−16.68, −9.36], *I^2^* = 32 %, *p* < 0.00001), general health (WMD = −7.80, 95 % CI: [−12.35, −3.25], *I^2^* = 62 %, *p* = 0.0008), body pain (WMD = −3.54, 95 % CI: [−6.82, −0.26], *I^2^* = 0 %, *p* = 0.03), vitality (WMD = −8.34, 95 % CI: [−14.31, −2.36], *I^2^* = 74 %, *p* = 0.006), social function (WMD = −6.44, 95 % CI: [−12.17, −0.72], *I^2^* = 71 %, *p* = 0.03), role emotional (WMD = −12.18, 95 % CI: [−16.46, −7.90], *I^2^* = 0 %, *p* < 0.00001), mental health (WMD = −6.79, 95 % CI: [−11.69, −1.88], *I^2^* = 72 %, *p* = 0.007).

**Table 2:** Meta-analysis of quality of life and results of heterogeneity analysis.

Item	Effect size (95 % CI)	p	*I^2^*	Source of heterogeneity	Effect size (95 % CI)^a^	p^a^	I^2^^a^
Physical function	−6.46 (−10.48, −2.44)	0.002	52 %	Chen	−4.77 (−8.1, −1.44)	0.005	0 %
Role physics	−13.02 (−16.68, −9.36)	<0.00001	32 %	‒	‒	‒	‒
General health	−7.80 (−12.35, −3.25)	0.0008	62 %	Chen	−5.49 (−8.8, −2.18)	0.001	0 %
Body pain	−3.54 (−6.82, −0.26)	0.03	0 %	‒	‒	‒	‒
Vitality	−8.3 4 (−14.31, −2.36)	0.006	74 %	Chen	−5.55 (−9.19, −1.91)	0.003	0 %
Social function	−6.44 (−12.17, −0.72)	0.03	71 %	Chen & Borland	−7.76 (−12.67, 12.84)	0.002	0 %
Role emotional	−12.18 (−16.46, −7.90)	<0.00001	0 %	‒	‒	‒	‒
Mental health	−6.79 (−11.69, −1.88)	0.007	72 %	Chen	−4.67 (−7.66, −1.69)	0.002	0 %

a Analysis results after removing the source of heterogeneity.

According to the *I^2^* values, either a random effects model or a fixed effects model was employed. The authors found that the study by Chen et al. was the main source of heterogeneity in the assessment of quality of life. Excluding this study did not affect the results but reduced the heterogeneity to 0 % (Table 2).

## Discussion

The present study demonstrated that patients with atrial fibrillation showed improved cardiorespiratory fitness and enhanced quality of life following exercise. Additionally, they experienced significantly lower recurrence rates after ablation compared to those who did not engage in exercise. These findings align closely with previous research in this area.

### Cardiovascular fitness and cardiovascular event

Cardiorespiratory function is recognized as a prognostic indicator for patients with coronary artery disease and heart failure.^5^ Evidence strongly supports the improvement of cardiorespiratory function in patients with atrial fibrillation through exercise rehabilitation.^6^, ^7^, ^8^, ^9^, ^10^, ^11^ However, current randomized controlled trials have not consistently shown improvements in mortality or readmission rates among AF patients participating in exercise.^7^^,^^12^^,^^13^ Notably, most of these trials have involved rehabilitation periods of less than six months and follow-up durations limited to one year, potentially insufficient to demonstrate significant differences in cardiovascular outcomes.

This suggests that the impact of cardiac rehabilitation on cardiovascular outcomes in AF patients may depend on the duration of exercise intervention. Retrospective studies with longer follow-up periods and larger participant cohorts tend to show a positive effect of exercise on outcomes such as death, stroke, and hospital readmission.^14^^,^^15^ Garnvik et al. summarized findings from the Hunt atrial fibrillation cohort involving 1117 patients, revealing that higher physical and cardiorespiratory fitness levels were associated with reduced risks of all-cause mortality and cardiovascular death in AF patients.^16^

Thus, the authors hypothesize that exercise may enhance AF prognosis by indirectly improving cardiorespiratory fitness.

### Heart rate control

Atrial fibrillation impaired atrial contraction results in ineffective blood ejection and reduced cardiac output by 20 %‒30 %.^17^ The irregular and often rapid ventricular rate further decreases ventricular filling and stroke volume.^18^ This combination of factors can lead to symptoms such as palpitations and shortness of breath in patients with insufficient per-beat output. Effective heart rate control is crucial in managing AF. Both European and US guidelines recommend a lenient approach to heart rate control in patients with AF, with a target heart rate of < 110 beats/min.^3^^,^^19^ Previous studies have demonstrated that exercise significantly reduces resting heart rate in AF patients compared to control groups or pre-exercise levels.^8^^,^^12^^,^^20^ The mechanism behind this effect may involve the modulation of autonomic nervous system function through exercise.^21^ However, the reduction in heart rate is typically modest, averaging around 3‒5 beats/min. Given the importance of heart rate management in AF patients, exercise rehabilitation can complement pharmacological therapy by aiding in heart rate control.

### Catheter ablation

Catheter ablation is recommended in the 2020 ESC and 2023 American College of Cardiology/American Heart Association (ACC/AHA) Guidelines for the Diagnosis and Management of Atrial Fibrillation as a class I recommendation after failure of pharmacological rhythm control and as a class II recommendation as first-line therapy.^3^^,^^19^ Despite advancements in ablation techniques, such as contact force catheters and mapping systems, the long-term success rates for Atrial Fibrillation (AF) ablation remain suboptimal, with recurrence rates ranging from 20 % to 50 %.^22^

Reducing AF recurrence post-ablation is a significant challenge in the field. Innovations in ablation methods, such as low-voltage zone and Roter ablation and high-power short-duration ablation, may improve outcomes. Additionally, there is growing interest in exploring the role of exercise in reducing AF recurrence. For instance, Chen et al. conducted an RCT involving 60 patients who underwent cardiac rehabilitation with aerobic exercise for up to one year, reporting a higher rate of sinus rhythm in the rehabilitation group compared to controls (78.3 % vs. 69.4 %, *p* < 0.05).^23^ However, Kato et al., in a study involving 61 patients with persistent AF post-ablation, found no significant difference in recurrence rates between patients who received cardiac rehabilitation and those who received conventional therapy after six months (21.4 % vs. 25.8 %, RR = 0.83, 95 % CI 0.33‒2.1).^10^ The 2020 ESC Guidelines advocate for encouraging moderate-intensity exercise and physical activity to prevent the onset or recurrence of AF.^3^ While larger RCTs and prospective cohort studies are needed for stronger evidence, exercise remains a low-cost and potentially beneficial component of AF management. Clinicians are encouraged to actively recommend exercise to patients, as long as it does not compromise standard care.

### Left ventricle ejection fraction

The present study suggests that left ventricular ejection fraction improves in patients after exercise compared to before, but the limited number of included studies and heterogeneity present challenges in interpretation.^24^^,^^25^ The impact of exercise on LVEF in patients with heart disease remains inconclusive, lacking clear mechanistic explanations.

AF is prevalent in heart failure, and vice versa, with AF predisposing to HF and HF predisposing to AF.^3^^,^^26^ AF is common across different categories of HF with varying EF values: a study analyzing a Swedish heart failure cohort of 41,446 patients found AF prevalence rates of 65 % in HF with preserved EF (HFpEF), 60 % in HF with mid-range EF (HFmrEF), and 53 % in HF with reduced EF (HFrEF), with AF independently associated with increased mortality in all HF types.^27^ Data from the Framingham Heart Study and the CHARM study further underscore the elevated mortality risk associated with AF in both HFpEF and HFrEF patients.^28^^,^^29^ This highlights that AF's impact on HF outcomes is significant irrespective of EF and may be particularly detrimental in HFpEF. Thus, rather than solely focusing on EF, it is crucial to actively manage the underlying conditions, control symptoms, and prevent complications in patients with AF and HF.

### Quality of life

The SF-36 scale has been widely used to assess the quality of life in AF. According to previous studies, exercise can improve the quality of life in more general populations.^30^ A large number of studies have suggested that the quality of life in patients with AF improves after exercise.^8^^,^^31^ Improved quality of life after exercise in patients with AF may be related to a variety of factors, such as improved cardiorespiratory fitness, improved metabolic markers, a reduction in AF premature beat load, or the pleasurable experience of exercise itself.

### Exercise intensity

Some studies have reported a higher incidence of Atrial Fibrillation (AF) in athletes compared to the general population, suggesting that excessive exercise increases AF risk.^32^^,^^33^ The choice of exercise intensity during rehabilitation is therefore delicate and important for patients with AF. ESC guideline highlights that prolonged high-intensity exercise in athletes can elevate AF risk and recommend moderate-intensity exercise to mitigate this risk.^3^ Moderate intensity is typically defined as peak oxygen uptake between 40 % and 69 %, peak heart rate between 55 % and 74 %, reserve heart rate between 40 % and 69 %, and a subjective fatigue index of 12 %‒13 %.^2^ Several randomized controlled trials have compared different exercise intensities in patients with AF and found no significant differences between groups during follow-up periods.^12^^,^^34^ There are relatively few studies in this area, possibly due to fears about the potential dangers of high-intensity exercise. More clinical research is needed to identify the benefits and risks of different exercise intensities for patients with atrial fibrillation.

### Limitation

This research has some limitations. The first is unexplained heterogeneity. The method of eliminating studies one by one to test the results of meta-analysis is a common method of sensitivity analysis. By eliminating one by one, if the heterogeneity becomes insignificant after eliminating a certain study, which can be considered as the source of heterogeneity, but the authors can't exclude this study individually to get better research results. As the present research shows, the heterogeneity of meta-analysis is significantly reduced after excluding Chen's article. The authors can try to infer the possible sources of heterogeneity by studying the characteristics of this study. Chen's article is comparable to other documents in terms of intervention measures, exercise intensity, types of atrial fibrillation, rehabilitation cycle, etc., but Chen's article is of low quality and has high bias in random sequence, hidden distribution, blind method and other issues, which may be the source of heterogeneity. In addition, the research samples the authors included are relatively small, and the number of people included in the overall meta-analysis is only 631. In view of the limited sample size, these findings are preliminary and more random studies are needed. Thirdly, the present research mainly focuses on short-term results, which is mainly caused by the fact that the current RCT only focuses on short-term results. It will be beneficial to identify the influence of exercise rehabilitation on long-term cardiovascular events, hospitalization and mortality of patients with atrial fibrillation, which will enhance the correlation of clinical practice findings. This point needs to be explained by a longer follow-up study in the future.

## Conclusion

The authors conducted this meta-analysis of RCTs and found that exercise rehabilitation has benefits for patients with AF. Exercise can improve quality of life and possibly reduce recurrence. Although exercise intervention demonstrates efficacy in enhancing key indices of cardiorespiratory fitness, including VO_2_max, 6MWT performance, and maximal power, measures of cardiac function such as ejection fraction and heart rate remain largely unaffected.

## Data availability statement

All data are available within the text.

## Informed consent and patients’ details

All data in this study are derived from published papers, and no informed consent is involved.

## Ethnic committee information and study protocol number

This study involves no ethical issues; as a meta-analysis, it has no protocol registration information.

## Authors’ contributions

Zhen Zhang and Jialing He wrote the paper and performed statistical analysis. Yinlan Hu Shuyao Wang and Duan Luo rechecked the data and statistical results and also revised this paper. Bao Xu, Guijun He, Xianchen Yang screened the publications and extracted data. Hanxiong Liu and Jialing He performed quality assessment and made figures and tables. Hanxiong Liu provide idea and tools. Zhen Zhang submitted manuscript.

## Funding

The National Clinical Key Specialty Construction Project.

## Conflicts of interest

The authors declare no conflicts of interest.

## Appendix Supplementary materials

Image, application 1
