# Supplementary material for: Cardiopulmonary fitness and quality of life improvements in atrial fibrillation patients following exercise-based cardiac rehabilitation: meta-analysis of randomized control trials
Source: Clinics (Sao Paulo). 2026 Mar 20;81:100907. doi: 10.1016/j.clinsp.2026.100907 (PMC13019935; doi:10.1016/j.clinsp.2026.100907)

**CLINICS-D-25-00482_ Supplementary Material**

**Supplementary Figure 1** The risk of bias. The quality of the selected studies is assessed according to the Cochrane criteria.


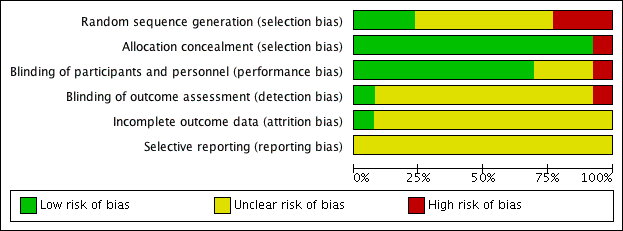

Supplement: Supplementary file 1 [file mmc1.doc]
